# Cancer Stem Cells, Bone and Tumor Microenvironment: Key Players in Bone Metastases

**DOI:** 10.3390/cancers10020056

**Published:** 2018-02-20

**Authors:** Ilaria Roato, Riccardo Ferracini

**Affiliations:** 1 Center for Research and Medical Studies (CeRMS), A.O.U. Città della Salute e della Scienza, Turin 10126, Italy; 2 Department of Surgical Sciences (DISC), Orthopaedic Clinic-IRCCS A.O.U. San Martino, Genoa 16132, Italy; riccardoferraciniweb@gmail.com

**Keywords:** bone metastases, cancer stem cells, osteotropism

## Abstract

Tumor mass is constituted by a heterogeneous group of cells, among which a key role is played by the cancer stem cells (CSCs), possessing high regenerative properties. CSCs directly metastasize to bone, since bone microenvironment represents a fertile environment that protects CSCs against the immune system, and maintains their properties and plasticity. CSCs can migrate from the primary tumor to the bone marrow (BM), due to their capacity to perform the epithelial-to-mesenchymal transition. Once in BM, they can also perform the mesenchymal-to-epithelial transition, allowing them to proliferate and initiate bone lesions. Another factor explaining the osteotropism of CSCs is their ability to recognize chemokine gradients toward BM, through the CXCL12–CXCR4 axis, also known to be involved in tumor metastasis to other organs. Moreover, the expression of CXCR4 is associated with the maintenance of CSCs’ stemness, and CXCL12 expression by osteoblasts attracts CSCs to the BM niches. CSCs localize in the pre-metastatic niches, which are anatomically distinct regions within the tumor microenvironment and govern the metastatic progression. According to the stimuli received in the niches, CSCs can remain dormant for long time or outgrow from dormancy and create bone lesions. This review resumes different aspects of the CSCs’ bone metastastic process and discusses available treatments to target CSCs.

## 1. Introduction

Due to advances in early diagnosis and cancer treatments, the survival of cancer patients has improved over the last two decades. As a negative consequence, the probability of cancer patients developing metastasis has increased. Bone is one of the most common sites of metastases, since some tumors show a marked osteotropism [1]. The propensity of some tumor to metastasize to bone depends on many factors such as the expression of adhesive molecules and bone markers on the surface of tumor cells (i.e., vitamin D receptor, RANKL, RUNX2, αVβ3, etc.) [2,3]; the expression of a set of genes associated with bone metastases [4,5,6,7,8,9,10,11,12,13,14,15,16,17,18,19,20,21]; and the capability of performing the epithelial–mesenchymal transition (EMT) of carcinomas [2,3,22,23,24,25,26,27,28,29,30,31,32,33,34,35,36,37,38,39,40,41,42,43,44,45,46,47,48,49,50,51,52,53,54,55,56,57,58,59,60,61]. In the bone microenvironment, cancer cells disrupt the physiological balance between bone resorption and formation, leading to lesions. Indeed, the interaction between cancer and bone cells causes the disarrangement of the bone matrix, disrupting the bone micro-architecture and impairing the bone function [62,63,64]. 

Literature reports that bone marrow (BM) is an appealing site for tumor cells and particularly for cancer stem cells (CSCs), which are able to resist treatments, such as chemo-radiotherapy. Indeed, once CSCs reach BM, they can remain dormant or cause bone metastases, long after the primary tumor has been surgically removed from patients [65,66]. CSCs have been isolated from many tumors and have been characterised for the expression of markers according to their different origin: the most representative markers with their functions are reported in Table 1. In this work, we focus on the dialogue among CSCs, bone and the tumor microenvironment, and the consequent formation of bone metastases.

## 2. Cancer Stem Cells (CSCs) Have a Pivotal Role in Tumor Heterogeneity

Tumor mass is constituted by a heterogeneous group of cells responsible for its formation and maintenance. This cell heterogeneity of many cancer types derives from a hierarchical organization that resembles one of the tissue of origin [80,107]. According to the hierarchical model, in the primary tumor at the apex of the hierarchy there are cells showing stem cell-like properties, the so-called CSCs. The phenotype and function of cancer CSCs appear to be equivalent to normal stem cells, but they harbour oncogenic mutations. CSCs possess high regenerative properties: self-renewal, and the ability to modify the response to stress conditions and thus survive in hostile conditions [108,109]. Date in literature, deriving from mouse models, provides genetic evidence that primary brain, colon and skin cancers comply with the hierarchical organization [110,111]. Also, clinical data confirm this hierarchical model; indeed, cell populations isolated from primary tumors using stem-cell marker genes can generate tumors when transplanted into mice [112,113,114]. Stem-cell properties are mainly maintained by four known transcription factors, such as octamer-binding transcription factor 4 (Oct4), Nanog, SRY-Box 2 (Sox2) and Kruppel-like factor 4 (Klf4) [115,116,117,118] and by different growth factors, such as epidermal growth factor (EGF) and fibroblast grow factor (FGF) [119,120]. To maintain the pluripotent stem-cell state Oct4 and Sox2 bind together forming a heterodimer, before linking to different sites on Klf4. Then, the Oct4/Sox2/Klf4 complex binds Nanog promoter [121]. Among these transcription factors, Oct4 is the main regulator, indeed the knock out of Oct4 expression dramatically reduces Sox2 and Nanog expression [122]. Moreover, Oct4 controls its expression: its increase inhibits the transcription of the *Nanog* gene and thus reduces Oct4 expression [123]. This work demonstrates that a delicate balance among these transcription factors influence the stem-cell phenotype [124].

Tumor heterogeneity also depends on the presence of various genetically related subclones, which compete with each other thanks to their different characteristics and evolve during tumor progression, allowing the acquisition of genetic instability and somatic mutations that favour one tumor clone over others [125,126,127]. Genome-sequencing studies have shown more similarities than differences between primary tumors and metastasis, suggesting that most of the genetic mutations required for metastasis are already present in the primary tumors [128,129]. Lastly, the environment where cancer cells reside activates epigenetic mechanisms, such as epigenetic reprogramming, which induces the stem-cell state [130].

## 3. EMT: A Key Step in CSC-induced Bone Metastases

EMT is a crucial step in tumor progression and has an important role during cancer invasion and metastasis. The importance of EMT for the induction of stem characteristics has been demonstrated in different carcinomas [131,132]. In immortalized human mammary epithelial cells, induction of an EMT results in the acquisition of mesenchymal traits and in the expression of stem-cell markers [133]. Some recent studies in transgenic mouse models showed that CSCs undergo EMT and initiate a tumor process [134,135]. These data were confirmed in clinical studies, showing the presence of tumor cells with CSCs capabilities in BM of breast cancer patients [136]. CSCs exist both in epithelial and mesenchymal states [133,137]: they perform EMT and express epithelial markers in order to promote migration from the primary tumor to secondary organ, such as BM, where they perform a mesenchymal-epithelial transition (MET), expressing markers that allow them to proliferate and initiate secondary lesions. The EMT transition is also a mechanism involved in bone metastasis formation [138]. Different studies reported that growth factors, such as transforming growth factor-β (TGFβ) and insulin-like growth factor (IGF), produced or released from the bone microenvironment, are potent effectors of EMT and can stimulate the formation of bone metastasis [139,140,141]. 

CD44 is an adhesion molecule that binds to the extracellular matrix through hyaluronan [142], increases the expression of the receptor activator of nuclear factor (NF)-κB ligand (RANKL) in stromal cells of BM, and promotes osteoclastogenesis [143,144]. CD44 has been recognized as one of the cell surface markers of CSCs in different tumors [145,146], and its expression is linked to an enhanced ability of CSCs to metastasize [147]. For instance, CD44 is significantly expressed by breast cancer cells, where it promotes invasion and adhesion to BM [148], and breast CSCs were initially identified as CD44^+^CD24^−^ cells [70,149]. Clinical studies suggested a positive correlation between CD44 expression and bone metastasis [136,150]. Hiraga et al. demonstrated that breast CSCs’ migration, invasion and their ability to form bone metastasis in nude mice were inhibited by the down regulation of CD44 [151]. In a human-in mouse model, we previously showed that breast CD44^+^CD24^−^ CSCs were present in the primary tumor, with a mesenchymal phenotype allowing them to migrate towards bone. After reaching the bone, in order to proliferate they performed a MET transition acquiring an epithelial phenotype; indeed, we detected CD44^−^CD24^+^ cells in bone. Once CD44^−^CD24^+^ cells collected by bone lesions were re-injected in mice, they formed new tumor masses, with a heterogeneous population, constituted mainly by CD44^+^CD24^−^ cells [4]. Our data confirm a previously reported work of Liu et al. demonstrating a transition between epithelial and mesenchymal states of breast CSCs [5]. Also, Chaffer et al. demonstrated that CD44^low^ breast-cancer cells can spontaneously convert into CD44^high^ CSCs both in vitro and in vivo [130]. The capability of the breast metastatic CD44^−^CD24^+^ cells isolated from bone to generate new heterogeneous tumors with a high percentage of CD44^+^CD24^−^ CSCs is consistent with a phenotypic plasticity of these cells, that allow metastatic cells to regain a tumor-initiating capacity. This transition between different states observed in CSCs can be considered a stochastic transition, and can be explained by the quantitative Markov model of cell-state interconversion, where any subpopulation of cancer cells returns to equilibrium, with the phenotypic proportion of the primary tumor over time. Thus, by contrast with the hierarchical model and according to the stochastic one, breast cancers can be constituted by discrete populations that randomly perform transitions between states without increasing their proliferation rate, simply to reach a progressive equilibrium proportion [6,7].

## 4. Role of CXCL12–CXCR4 Axis in CSC-Tumor Microenvironmet Crosstalk

CSCs seem to be quite pleiotropic in the expression of receptors in response to different microenvironmental stimulation, and thus they can modify their expressions in response to the level of ligand and move towards a secondary organ according to a chemotactic gradient. One of the main chemotactic stimulus involved in the regulation of trafficking of normal and CSCs is represented by the C–X–C motif chemokine 12 ligand (CXCL12) and C–X–C chemokine receptor type 4 (CXCR4) axis [8]. Indeed, CXCR4 is expressed not only in normal stem cells in different organs, but also in several tumors [9]. The CXCR4–CXCL12 axis is involved in cancer-cell-tumor microenvironment interactions and it is one of the mechanism involved in bone metastases formation [10]. Indeed, bone-seeking breast cancer cells express high levels of CXCR4, which is associated to cancer-cell stemness [11,136].

CXCL12 is highly expressed in bone, lymph nodes and lungs, CSCs expressing CXCR4 are attracted by these organs [12,13]. CSCs recognize chemokine gradients toward BM, like the gradient followed by hematopoietic stem cells (HSCs) and leukocytes to migrate to the bone [14]. Indeed, both mesenchymal stromal cells and osteoblasts (OBs), in BM, constitutively express CXCL12 [15], and they help the entrance of CSCs expressing CXCR4 into the bone microenvironment. In non-small cell lung cancer (NSCLC), the expression of the CXCR4 receptor has been associated with the maintenance of stemness of chemoresistant CSCs both in cell lines and primary tumors [16,17]. CXCL12 has been proven to induce EMT in different tumors [18,19], such as NSCLC, where a subset of CD133^+^CXCR4^+^ EpCAM^−^ CSCs could be directly generated through EMT activation [17]. Indeed, we demonstrated in a humanized murine model that CD133^+^CXCR4^+^ EpCAM^−^ CSCs were endowed with an enhanced bone tropism [20]. The ability of CD133^+^CXCR4^+^ CSCs to originate frank metastases when injected in humanized mice implanted with human bone stresses the prominent relevance of a conductive tumor microenvironment (with a pre-metastatic niche) to support tumor metastasis [139,140]. The over-expression of CXCR4 and CXCR7 by breast and prostate cancer cells increases their vascular ability and bone colonization in mouse models [21,152]. CXCR4 is also essential for maintenance of renal cell carcinoma-initiating cells [153].

## 5. Tumor Microenvironment and CSCs

The tumor microenvironment is composed of non-malignant cells such as endothelial cells, fibroblasts, immune cells and a non-cellular matrix. Both the cellular and non-cellular components form the tumor stroma, which dramatically changes during the tumor progression, influencing tumor growth and chemoresistance [154,155]. The tumor microenvironment releases different factors, it is able to protect CSCs against the immune system, and it maintains CSCs’ properties and plasticity [156]. Furthermore, the primary tumor stroma also seems to select for organ-specific seeding traits. For example, cancer-associated fibroblasts (CAFs), in breast-cancer stroma, produce CXCL12 and IGF-1, which select Src hyperactive cancer clones, and promote bone metastases [157,158]; in NSCLC, stimuli from CAFs in the tumor microenvironment could dictate de novo creation of the CD133^+^CXCR4^+^ CSCs subset, directly linked to EMT induction with the acquisition of increased dissemination [20]. In colorectal cancer, CAF release hepatocyte growth factor, which stimulates the self-renewal of CSCs through a β-catenin-dependent mechanism [159] and promotes the reprogramming of colorectal cancer progenitors into CSCs [160]. Moreover, CAFs secrete specific cytokines and chemokines upon chemotherapy treatment, such as IL-17A, that stimulates colorectal CSC self-renewal and invasion [161]. This last observation indicates that chemotherapy induces a remodelling of the tumor microenvironment and thus contributes to promote CSC proliferation [162].

## 6. CSCs’ Dormancy in the Niche

**Figure 1:**
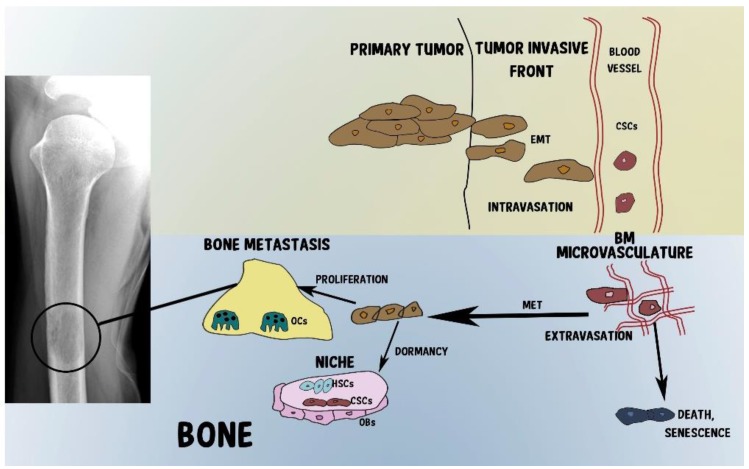
Cancer stem cells’ (CSCs) steps toward bone metastasis. At the primary tumor, cancer cells grow and can perform epithelial-to-mesenchymal transition (EMT), which confers them with invasive properties and consequent intravasation in the circulation. Once they have reached the BM microvasculature, CSCs can undergo senescence and cell death, or colonize the BM, regaining their original epithelial phenotype through a mesenchimal-to-epithelial transition (MET). Then CSCs can enter into a quiescent state (dormancy) in the BM niches, where osteoblasts (OBs) contribute to dormancy, or they can proliferate, releasing factors activating osteoclasts (OCs) and forming bone lesions.

CSCs have the capability to grow as spheres, and thus they can easily enter and disseminate in blood circulation. Once they have reached a secondary organ, such as BM, they can remain silent or undergo asymmetric division, allowing the maintenance of the CSC population as well as the expansion of differentiated cancer cells, which represent the full spectrum of the original tumor heterogeneity and maintain secondary lesions [163,164,165] (Figure 1).

CSCs metastasize to the BM, localizing in HSC niches, creating the so-called pre-metastatic niches, which are anatomically distinct regions within the tumor microenvironment that govern metastatic progression [166,167]. OBs are constituent of the niches and maintain HSC by secreting growth factors such as stem-cell factor (SCF), CXCL12 and angiopoietin [168,169]. The importance of OBs in supporting the metastatic niche has been demonstrated in prostate cancer, where in an in vivo model the ablation of OBs significantly decreased the presence of disseminated cancer cells in the BM [170]. Compelling evidence from Shiozawa et al. reported that disseminated prostate cancer cells compete with HSC for niche support, where they can be remobilised in the circulation by the same molecules that induce HSC [170]. OBs also control long-term dormancy and support the cell survival of CSCs [171]: they exert a protective role for CSCs from the immune system, preserve their phenotypic plasticity, and promote their dormancy or metastatic potential.

Dormancy depends on different exogenous factors released by several BM stromal cells and the matrix: for instance, OBs express cadherins which allow bonds with CSCs [172] and the bone matrix [173]; annexin II expressed by OBs and endothelial cells plays a critical role in niche selection [174]. OBs also secrete factors such as growth arrest specific 6 (GAS6), which binds to the receptor tyrosine kinase AXL, activating it and thus promoting tumor cell dormancy [175]. Osteopontin induces mesenchymal stem cells in the tumor microenvironment to differentiate into CAFs, which promote cancer growth and can be educated to release periostin in the metastatic microenvironment [22,23]. Periostin is a critical factor promoting the stemness of CSCs and the CSCs’ niche via the IL-6/STAT3 signaling pathway [23,24], and the expression of IL-6 has also been associated with the dormancy phenotype. Recent data reported that dormancy could also be an intrinsic feature of a subpopulation of cancer cells that have arisen in a hypoxic microenvironment, which can remain dormant initially and later on be responsible for disease relapse [25].

Perivascular niches are important for the maintenance of both HSCs [26] and CSCs [27]. Indeed, endothelial cells in the mature microvascolature express thrombospondin-1 and other factors which maintain breast CSCs dormant [28]. On the other hand, endothelial cells in the sprouting neovasculature produce tenascin C, fibronectin and other factors that induce the formation of different perivascular niches and accelerate CSCs’ outgrowth [28]. Thus, it is likely that the dynamic of the niche components, rather than spatial localization, regulates the quiescence or the proliferation of CSCs.

## 7. Outgrowth from Dormancy

CSC-niche crosstalk is fundamental for outgrowth from dormancy and is affected by the bone microenvironment, which can remove crucial signals able to maintain cells dormant in the metastastic niche. Indeed, the balance between dormancy and CSCs’ re-activation depends on microenvironmental factors, which contribute to the outgrowth from the dormancy of CSCs, and their interactions with stromal cells in bone [29]. For instance, stromal cells can be activated by dormant breast CSCs to release niche extracellular matrix components, such as Periostin and Tenascin C, which in turn activate the crucial stem-cell signalling pathways such as Wnt, Nanog and Oct4, fostering proliferation and outgrowth from the dormancy of CSCs [23,172]. In the tumor microenvironment, fibronectin and type I collagen were reported to stimulate ERK/MAPK and Src driven proliferation, shifting tumor cells from dormant to proliferative via β1-integrin stimulation [30,31,32]. Specifically, in the BM, multiple myeloma cell dormancy has been described as a reversible state, which is switched on by OBs and switched off by OCs remodelling the endosteal niche [171]. Indeed, OCs remodel the surface of the endosteal niche releasing dormant myeloma cells and favouring their outgrowth from dormancy [171]. A similar mechanism of OC-mediated escape from dormancy has been reported in breast-cancer cells that aberrantly express the vascular cell-adhesion molecule 1 (VCAM-1), recruit α4β1 OC precursors by engaging in VCAM-1/α4β1 binding to increase Oct activity, and stimulate tumor growth [166]. The physiological bone remodelling, through the release of different molecules, provides a fertile soil for the growth of tumor cells, such as TGF-β, IGF I and II, platelet-derived growth factor (PDGF), fibroblast growth factor (FGF), and bone morphogenetic proteins (BMPs) [33,34]. It is also responsible for the high extracellular Ca^2+^ concentration, which can bind to calcium-sensing receptors and stimulate PTH-related peptide (PTHrP) in re-activated CSCs, promoting tumor-cell proliferation and survival [35,36]. Moreover, physical factors such as acid pH, hypoxia and high extracellular calcium concentration contribute to CSCs’ awakening and, thus, metastasis formation in breast cancer [166]. Proliferating tumor cells can secrete prostaglandins, PTH, activated vitamin D, IL-6 and tumor necrosis factor (TNF), leading to an increase in RANKL expression on OBs and BM stromal cells [34], which stimulates OC numbers, survival and activity. Among the factors and pathways that regulate the progression of CSCs to bone metastases there are also VCAM1, tumor-induced OC miRNAs and Jagged1 [37,38,39]. In particular, Jagged1 is a ligand of Notch, one of the most important downstream mediators of the pro-metastatic TGF-β, that directly activates OC differentiation and promotes tumor growth, stimulating IL-6 production by OBs [40].

Recent data reported that tumor-derived microvescicles, such as exosomes, are also involved in metastasis formation. They contain integrins, which mediate the homing of metastastic cells to specific secondary organs [41], and they can transfer oncogenic proteins and nucleic acids, modulating the fate of tumor cells and target organs [42,43,44].

## 8. Targeting CSCs to Block Bone-Metastasis Formation

Since CSCs play a fundamental role in promoting bone metastases, they represent a target for novel or combined anti-cancer therapies. Indeed, targeting and eliminating CSCs is an approach largely envisioned to avoid cancer dissemination, disease relapse and thus the development of bone metastases. Methods to target CSCs may be represented by drugs inhibiting CSC-specific signalling pathways, compounds targeting alterations in CSC metabolism, methods to induce differentiation or a loss of stemness, and immunotherapy directed at CSC markers [45].

Different groups have focused their efforts on studies aimed at discovering new targeted CSCs therapies. For instance, BR-DIM, a cruciferous vegetable metabolite added to the prostate cancer cell cultures, was proved to inhibit self-renewal ability of CSCs [46], suggesting that this treatment may induce CSC terminal differentiation and prevent therapeutic resistance.

Another successful approach has recently been reported by Cuyas et al. for aggressive forms of breast cancer: they showed that in breast cancer, a dysregulation of the OPG/RANK/RANKL signalling axis is present and is associated with the presence of CSCs’ highly expressing RANK, residing in the pre-metastatic niche and able to initiate metastasis including bone lesions [47,48,49]. Moreover, in these tumors, bone metastases are also promoted by the low levels of OPG and high levels of RANKL. It is known that treatment with denosumab, an anti-RANKL antibody, reduces the mammosphere-forming ability in BRCA1-mutated epithelial cells, in triple negative and in HER2-overexpressing breast-cancer cells, supporting the notion that tumor and cancer initiating cells have hyperactive RANKL signalling in primary and metastatic sites such as bone. Moreover, the combination with metformin, an anti-diabetic molecule, with denosumab, showed a synergistic action of the two drugs in reducing breast CSCs [49].

Our group showed that renal CSCs expressing CD105 and c-MET can directly form osteolytic bone lesions, and a selective c-MET inhibitor treatment abrogated bone metastases’ development. Those data suggest that c-MET expression on renal CSCs drives renal cancer progression to bone, thus HGF/c-MET signalling is relevant in the metastatic bone process induced by these CSCs [50]. Based on previous studies on breast cancer reporting that CD44^+^ CSCs expressed also the oncogene c-MET [114], and its inhibition blocked bone metastases from breast cancer [51], we tested a selective c-MET inhibitor. We demonstrated it was able to inhibit the stimulatory activity of renal CSCs on OCs, likely interfering with paracrine factors produced by CSCs, which promoted OC bone resorption. On the other hand, we observed that it stimulated OBs, suggesting us to conclude that this inhibitor exerted both effects on bone cells and CSCs.

In addition, inhibitors of CXCR4, such as CTCE-9908, a small peptide analogue of SDF-1, have been demonstrated to be effective in preventing metastatic dissemination in different cancer models such as breast, oesophageal and prostate [52,53,54,55], by acting both on CSCs and on proliferating tumor cells. In particular in NSCLC, CXCR4 targeting was able to counteract the chemotherapy-induced metastatic spread of chemoresistant fractions of CD133^+^CXCR4^+^ CSCs, pointing at combination therapy with CXCR4 inhibitor as an attractive novel strategy to improve neo-adjuvant and adjuvant therapy for these tumors [20]. In a prostate-cancer model, the use of a neutralizing antibody to CXCR4 also reduced the growth of prostate cancer cells injected intra-tibia and the subsequent formation of bone metastases [13].

Other possible pathways of interest include Akt activation and Erk signalling [8,56], which may be up-regulated in CSCs in comparison with the bulk-tumor population and responsible for enhanced CSC survival. In addition, new small molecule inhibitors are under development; they target signalling pathways and transcription factors prevalent in CSCs but not in normal cells [57]. Moreover, CSC-targeted interventions, combination therapies could also target simultaneously several types of cells and pathways, such as bulk-tumor cells, CSCs in the niche, tumor-associated macrophages, tumor microenvironment or the adaptive immune system [45].

Lastly, CSC markers can be considered as immune targets [58], but examination of CSC markers is needed since some markers may identify both normal stem cells and CSCs, such as Trop2 and α6 integrin [59,60], causing adverse events in clinical trials.

## 9. Conclusions

The importance of targeting CSCs to avoid tumor metastases has emerged as a prominent concern. Thus, an integrated approach with the development of ex vivo and in vivo models should lead to further characterization of the CSCs. High-resolution imaging technology together with the identification of stromal markers will improve our understanding of CSCs in order to more accurately recapitulate the niche of tumorigenic cells and address novel mechanisms that operate in CSCs, especially during tumor progression. Moreover, continuous investigation of CSC markers is needed, since some markers may identify both normal stem cells and CSCs and cause adverse events in clinical trials. Lastly, many efforts are also advocated on the discovery of novel procedures to isolate, identify and enrich for CSCs, with the final goal of monitoring the disease in terms of prevention of tumor progression and resistance to treatments.

## Figures and Tables

**Table 1:** Cancer stem cell markers.

Marker	Family	Function	Tumors	Effects	References
CD44	Cell surface HA-binding glycoprotein	Tissue remodeling, adhesion of cell-matrix, and cell migration	Breast, Prostate, Liver	Aggressive phenotype, Tumor progression, Stemness phenotype, Bone metastasis	[4,67,68,69,70]
E-cadherin	Type I transmembrane protein	Maintain normal cell structure, cell polarity and integrity	Prostate, Breast, Brain	Stemness gene expression, Tumor progression, Invasion and metastasis, Theraphetic resistance	[71,72,73]
CD166	Immunoglobulin superfamily of cell adhesion molecules (Ig-CAMs)	Intercellular adhesion, leukocyte extravasation, T cell activation and proliferation, and stabilization of the immunological synapse	Lung	Cellular proliferation, Stemness phenotype	[74,75]
EpCAM	Epithelial cell adhesion molecule	Wnt-beta-catenin signaling	Liver, Prostate	Tumor progression, Invasion and metastasis, Therapheutic resistance	[76,77]
ABCB5	ATP-binding cassette sub-family B	Drug efflux transporter	Melanoma, Breast, Colorectal, Liver	Tumor progression, recurrence, Therapeutic resistance, Metastasis, Invasion	[78,79,80,81,82]
ABCG2	ATP-binding cassette (ABC)	Drug efflux transporter	Breast, Prostate, Liver	Stem cell phenotype, Proliferation, Migration, Therapeutic Resistance	[83,84,85]
ALDH	Detoxifying enzyme	Proliferation	Breast, Lung, Brain, Colon, Liver, Prostate, Bladder, Ovarian, Renal	Tumor progression, Self-renewal capacity,	[86,87,88,89,90,91,92]
CD133	Transmembrane protein	Proliferation, differentiation and self-renewal	Gastric, Lung, Liver, Colon, Renal, Prostate, Pancreatic	Tumor progression, Stemness gene expression, Bone metastasis	[20,93,94,95,96,97]
CD13	Membrane glycoprotein	Aminopeptidase N	Liver	Invasion, Angiogenesis, Proliferation	[98,99]
CD90	Glycosylphosphatidylinositol-anchored glycoprotein	Cell-cell and cell-matrix interactions	Liver, Breast, Lung	Invasion, Tumor progression	[100,101,102]
CD105	Type I membrane glycoprotein, TGF beta receptor complex	Angiogenesis, Mesenchymal Stem cell marker	Renal, Breast, Liver	Initiating metastatic process, Stemness gene expression, Migration, Bone metastasis	[50,103,104,105,106]
